# Alimentation Impact of Treatments of 254 Oropharyngeal Cancers (1998–2003)

**DOI:** 10.5402/2011/609517

**Published:** 2011-05-14

**Authors:** Guillaume Buiret, Clémentine Daveau, Guillaume Landry, Carole Colin, Jean-Christian Pignat, Marc Poupart

**Affiliations:** Service d'ORL, Centre Hospitalier Universitaire de la Croix Rousse, 103 Grande-Rue de la Croix Rousse, 69317 Lyon Cedex 04, France

## Abstract

*Objective*. To analyze the functional impact of the various possible treatments of oropharyngeal squamous cell carcinomas to find the main prognostic factors of dysphagia induced by these treatments. *Patients*. Clinical data from 254 patients treated for squamous cell carcinoma of the oropharynx between 1998 and 2003 were retrospectively analyzed. A multivariate model enabled us to evaluate the role of each potentially harmful factor on swallowing. *Main Outcome Measures*. The significant factors influencing the consumption of liquid, pasty, and normal food were the same: the initial T stage and the type of treatment. *Conclusion*. Whatever the possible and selected treatment was, the impact on the functional capacities, and thus, the quality of life of the patients was considerable. Even though we could not significantly demonstrate exclusive radiotherapy caused more long-term undesirable effects than surgery followed by radiotherapy, our daily practice has shown that we should favour the latter.

## 1. Introduction

Oropharyngeal cancers account for a major proportion of cancers of the upper aerodigestive tract. In 1995, the incidence in France was 3083 cases [1]. The prognosis remains poor with overall survival at 5 years estimated at 30% [1]. The standard treatments for these cancers can be either surgery followed by radiotherapy eventually with concomitant chemotherapy or exclusive radiotherapy eventually with concomitant chemotherapy. In spite of the lack of methodologically solid comparative studies, no difference in terms of survival between these two options seems to exist [2]. 

A new challenge recently appeared, certainly less important than survival, but important all the same: the quality of life of patients among whom dysphagia seems to be a very important criterion [3]. Some treatments may be too detrimental in particular with regard to swallowing. In the same way, the recently developed concept of therapeutic utility (measurement of the preference of patients for a health condition) is raised: will a patient prefer to live for three years with normal eating ability or for four years with exclusive enteral feeding? 

The aim of our study was to evaluate the short-, medium-, and long-term (five to 10 years) feeding abilities of patients treated for oropharyngeal cancer to determine prognostic factors for feeding difficulties.

## 2. Material and Methods

### 2.1. Selection of Patients' Records

After consulting the Medical Information Department database of the Croix-Rousse Hospital, 252 consecutive patients treated between January 1st, 1998 and December 31st, 2003 for oropharyngeal squamous cell carcinoma were selected.

### 2.2. Possible Treatments

If patients were operable, they were offered surgical treatment (tumor and lymphadenectomy) followed by radiotherapy (50–54 Gy in 5 to 6 weeks) sometimes potentiated by cisplatin according to the pathology results (more than 3 metastatic nodes, extracapsular extension, endovascular metastases, or perineural infiltration). The surgery procedures [4, 5] were transoral approach, submandibular approach (lateral oropharyngectomy, hyosubglossoepiglottectomy [6]), or transmandibular approach (mandibular swing or pharyngectomy with mandibulectomy). During surgery, a nasogastric tube was inserted, and tracheotomy was performed except in the case of small tumors treated by the transoral approach. Most of the patients benefited from swallowing rehabilitation during their hospitalization, which in certain cases continued after return home or after admission to a rehabilitation institution. Unfortunately, this variable could not be exploited because of the lack of data. 

If operable patients refused surgery, they received exclusive radiotherapy (70 Gy in 6-7 weeks) generally potentiated by cisplatin. 

In the event of an inoperable tumour, and sometimes after induction chemotherapy, the patient received exclusive radiotherapy (70 Gy in 6 to7 weeks) generally potentiated by cisplatin. 

Radiotherapy fractionation was standard (2 Gy per session, 5 sessions a week); 2 lateral ports with 4 to 8 MV X-rays split at 40 Gy due to the spinal cord tolerance limit to 2 anterior lateral ports with X-rays and 2 posterior ports with 8 to 10 MeV elecron beams. The level IV regions were treated with mixed 4–6 MV X-Rays and 8 to 10 MeV elecron beams. Additional boosts were given for high-risk nodal areas (in cases of node involvement with extracapsular extension) either with 8 to 10 MeV electron beams.

### 2.3. Statistical Analysis

The following data were retrospectively collected: weight, swallowing ability (solids, pastes, liquids) according to doctors' and speech pathologists' notes, and the use of a nasogastric tube during the posttherapeutic followup (five to ten years). After exclusion of the patients initially treated with palliative care or palliative chemotherapy (including metastatic patients), functional tolerance to treatments was analyzed in order to determine prognostic factors for post-therapeutic swallowing difficulties and their repercussion on body weight. The time onset for alimentation abilities was the date of diagnosis. Factors which were thought to have a significant impact on swallowing were the sex, the age, the American Society of Anaesthesiology (ASA) score, previous cancer of the head and neck, the tumour, location in the oropharynx, the T stage, the N stage, the use of induction chemotherapy, the type of treatment, the reconstruction type, the existence of a postoperative complication, the dose of radiation to the tumour and the dose of radiation to the nodes.

- For the study of weight, a linear regression according to time was used with nested mixed models on repeated data.
- The binary variables of liquids, pastes, and solid foods were studied using a multivariate model of survival censored by intervals. Multivariate Cox regression models were used. The reference patient was by definition a 55-year-old man, ASA 1, with no past history of cancer who developed T1N0 cancer of the base of tongue who received no induction chemotherapy and was treated using pharyngectomy with mandibulectomy, without postoperative complications or postoperative radiotherapy. Such a patient does not exist but changing one (or more) parameters of the model gives a hazard ratio that allows comparisons to be made. An example is given in the “Results” section.

The same model was used for liquids, pastes, and solid foods. The analyses were carried out with R v2.6.1 software (R foundation).

## 3. Results

### 3.1. Population and Demography

The characteristics of the patients are presented in Table 1. Sixty-two patients had previously at least one cancer of the head and neck (24.6% of the patients). It consisted of salvage surgery for 32 patients (12.7%). 

One hundred and thirty-five patients still drank alcohol at the time of the diagnosis (68.5% of the data collected on this item), while 133 patients still smoked at the time of the diagnosis (65.6% of the data collected on this item). The mean cigarette consumption was 43.5 pack years.

At the time of diagnosis, 19 patients (7.5%) had one or more synchronous cancers. Four patients presented pulmonary metastases, and one patient had osseous metastases at the time of the diagnosis.

### 3.2. Treatments

The different types of treatment are presented in Table 2.

#### 3.2.1. Induction Chemotherapy

Eighty-one patients (32.1%) had induction chemotherapy, the majority with 5-fluorouracil/cisplatin because of inoperable tumours.

#### 3.2.2. Surgery

**(i) Surgery for the Tumour**

One hundred and twenty-two patients (48.4% of the patients) did not have tracheotomy, 25 patients (9.9% of total patients, 15.8% of operated patients) underwent prepared tracheotomy, and 70 patients (27.7% of total patients, 44.3% of operated patients) had tracheotomy. The mean duration of the tracheotomy was 2.2 weeks (ES 3.3 weeks), the mean time before postsurgical recovery of feeding was 1.8 weeks (ES 1.8 weeks), and the mean duration of postsurgical nasogastric tube was 2.7 weeks (ES 1.8 weeks).

**(ii) Reconstructive Surgery**

The loss of substance due to tumor surgery required a pectoralis major flap for 23 patients (18 mandibular swings, 4 pharyngectomies with mandibulectomy, 1 lateral oropharyngectomy) and a sternocleidomastoid flap for seven patients, all after lateral oropharyngectomy.

**(iii) Complications of the Surgery**

Forty-one patients (25.9% of the operated patients) had a surgery-related complication: one death from an unknown cause, cervical infection (9), bleeding (8), pharyngostoma (7), cervical hematoma (4), pneumonia (3), orostoma (2), fever of an undetermined cause (2), flap disunion (2), hepatic failure (1), acute delirium (1), and lymphorrhea (1). The last nine patients were transferred to the intensive care unit during the postoperative phase: five transfers were scheduled (two for dialysis for chronic renal failure, three patients with a heavy cardiac past history requiring close surveillance), and four whose stay was unscheduled (one pneumonia, one hemorrhagic hypovolemic shock, one hepatic failure, one cardiac failure).

#### 3.2.3. Radiotherapy

Table 2 presents the different types of radiotherapy and Table 3 the doses of radiation. Fifteen patients had local brachytherapy during postoperative external radiotherapy, four during exclusive external radiotherapy, and one patient without external radiotherapy (postoperative or exclusive).

### 3.3. Evaluation of the Impact of Operators

191 patients (75.8%) have undergone surgery. 167 patients (87.7%) were operated on by the same surgeon who also ensured the medical followup of 242 patients (96.0%). The data collecting is therefore very uniform. 

### 3.4. Functional Evolution

**Figure 1:**
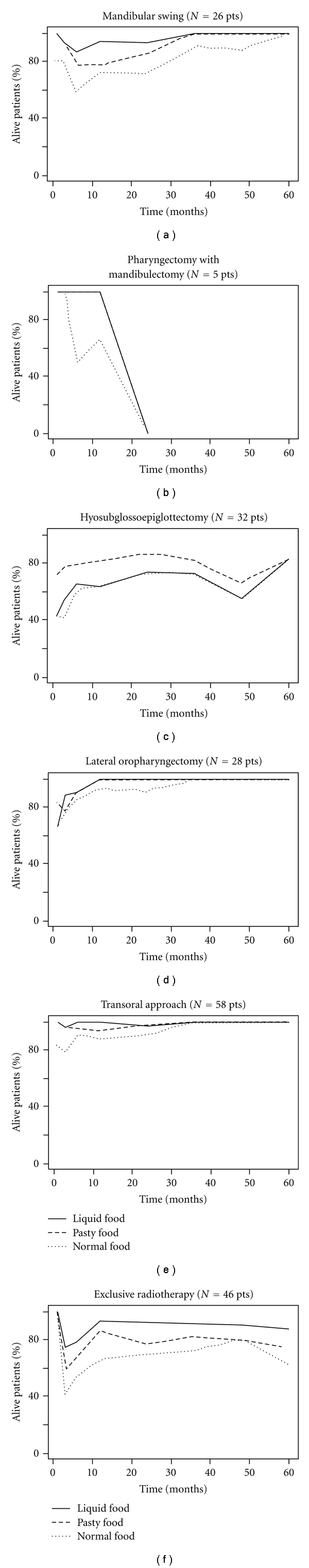
Swallowing evolution by type of treatments. Pts: patients. All pharyngectomy with mandibulectomy were performed on patients with recurrent tumours previously irradiated (salvage surgery).

**Figure 2:**
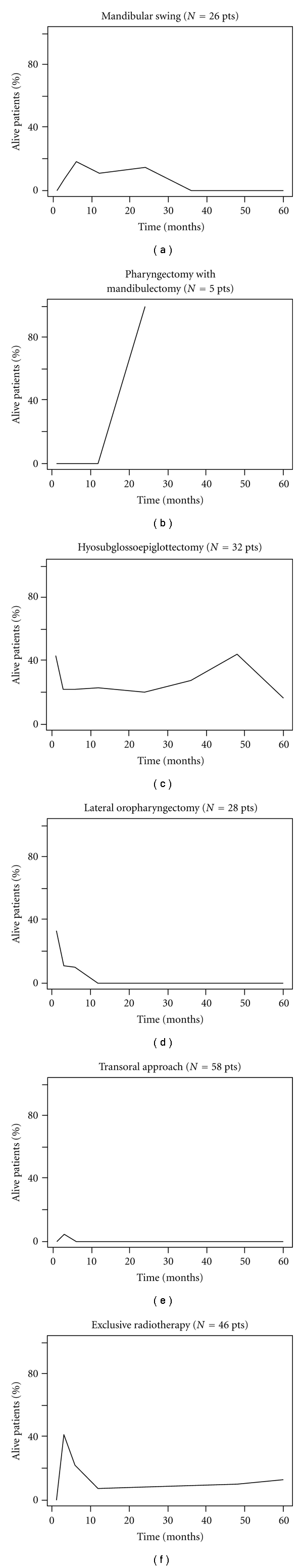
Evolution of nasogastric tube use by type of treatment.

Figure 1 presents the evolution of swallowing ability with time by type of treatment, and Figure 2 shows the evolution of the use of a nasogastric tube by type of treatment.

### 3.5. Study of the Weight

The mean weight at diagnosis was 64.9 kg. In the event of a history of cancer of the upper aerodigestive tract, the mean weight at diagnosis was significantly lower (−3.6 kg; *P* = .0173). Patients with a post-operative complication were significantly heavier (+1.2 kg, *P* < 10^−4^). Age, location of the oropharyngeal tumor, T stage, and N stage had no significant impact on weight at the time of diagnosis (*P* = .2282,.7536,.4524, resp.). 

The average weight loss was 1.56 kg/yr (*P* < 10^−4^) throughout the followup, and the type of treatment did not have a significant impact on weight loss (*P* = .634).

### 3.6. Prognostic Factors of Swallowing Disorders

Table 4 presents the results of the models' parameters. Because of the small numbers of patients in some categories (e.g. pharyngectomy with mandibulectomy), the confidence interval was large and did not allow us to prove any significant differences.

## 4. Discussion

In the international literature, patients' weight is seldom taken into account in the estimate of the functional results of treatments for cancer of the upper aerodigestive tract. However, denutrition is a predictive factor of tolerance to the treatment. According to our analyses, the treatments for oropharynx cancers, whatever they are and whatever the site of the oropharyngeal lesion is, caused a continuous, considerable (−1.56 kg/yr), significant and long-term (60 to 120 months of followup) weight loss in patients in an often already precarious nutritional state. However, correct feeding was rapidly possible for the majority of patients and remained possible with time. Significantly, factors influencing the ability to swallow liquids, pastes, and normal foods were generally the same: the initial T stage, induction chemotherapy, and the type of treatment. Because of the absence of anatomophysiological explanations for the harmful impact of induction chemotherapy on swallowing and the fact that this finding has not been described in other studies, it needs to be interpreted with caution, and other studies need to be conducted to rule out or to confirm this hypothesis. Putting aside the possible role of induction chemotherapy, the data of our study are in agreement with those already published. Indeed, for several authors [7–9], the functional outcomes of surgery followed by radiotherapy and radiochemotherapy were not significantly different except for those reported by Allal et al. [10] for whom the functional quality of life and abilities after exclusive radiotherapy were similar for T1T2 but significantly better for T3T4. Other authors showed the existence of predictive factors of good functional performances, but these factors varied from one study to another. For Zuydam et al. [11], swallowing was significantly better in the absence of radiotherapy (*P* < .001) and in the event of direct closure (*P* = .003), and speech was also significantly better in the event of direct closure. For Zelefski et al. [12], swallowing was significantly better for T1T2 than for T3T4. For Klozar et al. [13], the predictive factors of good swallowing were the type of surgery (in ascending order towards better swallowing: pharyngectomy with mandibulectomy, mandibular swing, and lateral oropharyngectomy) and location (in ascending order tonsil then base of tongue). This study should probably not be retained because of the illogical physiological character and the fact it is isolated from its conclusions. For Pauloski et al. [14, 15], swallowing depended especially on the volume of tissue removed. Lastly, for Van Cann et al. [16], only postoperative radiation decreased swallowing ability. In conclusion of the analysis of the literature, the functional results seemed better in the absence of postoperative radiation and of reconstructive surgery and in the event of small tumour size, which is perfectly logical from a physiological point of view. We could not show that certain treatments (in particular exclusive radiotherapy and transmandibular approaches) caused more serious swallowing disorders in the medium and long term. In our everyday clinical experience, the principal factor that has an impact on function, which proved to be relevant for all locations of upper aerodigestive tract cancers, is radiation and there seems to be a threshold level of radiation (60 Gy) above which deterioration of functional abilities is accentuated. However, this hypothesis could not be proved in this study probably because of the high doses of postoperative radiation, more often closer to 60–64 Gy than to 50–54 Gy (Figure 2). Patients had a dose of radiation that was higher than the requested dose, and it is advisable to carefully discuss doses of radiation with the radiotherapists.

## 5. Conclusion

Whatever the possible and selected treatment was, the impact on the functional capacities and, thus, the quality of life of the patients was considerable. Even though we could not significantly demonstrate that exclusive radiotherapy caused more long-term undesirable effects than surgery followed by radiotherapy, our daily practice has shown that we should favour the latter. Further prospective studies about long-term side effects of treatments of oropharyngeal malignancies should be conducted.

## Figures and Tables

**Table 1:** Characteristics of the patients.

Characteristics of the patients	
Age: mean (SD), yr	57.8 (10.9)
Sex: no (%)	
Male	223 (88.5)
Female	29 (11.5)
ASA stage at diagnosis: no (%)	
I	62 (24.6)
II	117 (46.4)
III	72 (28.6)
IV	1 (0.4)
BMI at diagnosis: mean (SD), kg/m²	22.7 (4.2)
Previous ENT cancer: no (%)	62 (24.6)
Relapse: no (%)	32 (12.7)
AJCC stage groups: no (%)	
I	14 (5.5)
II	28 (11.1)
III	43 (17.1)
IV	167 (66.3)
UICC T stage groups: no (%)	
T1	25 (9.9)
T2	81 (32.1)
T3	75 (29.8)
T4	71 (28.2)
UICC N stage groups: no (%)	
N0	105 (41.7)
N1	14 (5.5)
N2a	25 (9.9)
N2b	45 (17.9)
N2c	38 (15.1)
N3	25 (9.9)
UICC M stage groups: no (%)	
M0	247 (98.0)
M1	5 (2.0)

ASA: American Society of Anaesthesiology, BMI: Body mass index, AJCC: American Joint Committee on Cancer, and UICC: International Union against Cancer.

**Table 2:** Types of treatment.

Types of treatment	
*Induction chemotherapy: no *(*%*)	81 (32.1)
*Surgery: no *(*% of patients, * **% of operated patients**)	158 (62.7, **100**)
Submandibular approach	65 (25.8, **41.1**)
Hyosubglossoepiglottectomy	32 (12.7, **20.3**)
Lateral oropharyngectomy	28 (11.1, **17.7**)
Total laryngectomy extended in base of tongue	5 (2.0, **3.2**)
Transoral approach	58 (23.0, **36.7**)
Transmandibular approach	31 (12.3, **19.6**)
Mandibular swing	26 (10.3, **16.5**)
Pharyngectomy with mandibulectomy	5 (2.0, **3.2**)
Lymphadenectomy only	4 (1.6, **2.5**)
*Lymphadenectomy: no *(*% of patients, * **% of operated patients**)	157 (62.3, **99.4**)
Left	42 (16.7, **26.6**)
Right	48 (19.0, **30.4**)
Bilateral	67 (26.6, **42.4**)
*Postoperative complication: no *(*% of patients, * **% of operated patients**)	41 (16.3, **25.9**)
*Radiotherapy: no *(*%*)	173 (68.7)
Postoperative radiotherapy	112 (44.4)
With concomitant chemotherapy	46 (18.1)
Without concomitant chemotherapy	66 (26.3)
Exclusive radiotherapy	46 (18.1)
With concomitant chemotherapy	32 (12.7)
Without concomitant chemotherapy	14 (5.4)
Local curietherapy	15 (6.0)
*No postoperative radiotherapy: no *(*% of patients, * **% of operated patients**)	38 (15.1, **24.1**)
*Palliative chemotherapy: no *(*%*)	6 (2.4)
*Palliative care: no *(*%*)	9 (3.6)

**Table 3:** Doses of radiation by type of treatment.

	0 Gy	[20 Gy; 50 Gy]*	[50 Gy; 60 Gy]	[60 Gy; 70 Gy]	≥70 Gy	Total
Submandibular approach						
Hyosubglossoepiglottectomy	6	2	8	9	7	32
Lateral oropharyngectomy	2	2	6	17	0	28
Transoral approach	13	2	8	20	8	51
Transmandibular approach						
Pharyngectomy with mandibulectomy	5	0	0	0	0	5
Mandibular swing	3	0	4	18	1	26
Exclusive radiotherapy	0	0	4**	23	19	46

*[20 Gy; 50 Gy] means the patient received 20 Gy or more but less than 50 Gy. Every patient who received less than 50 Gy had previously undergone radiotherapy, and this dose was a complementary dose.

**indicates patients who stopped radiotherapy prematurely because of intolerance.

**Table 4:** Statistical study of swallowing.

Factors	Liquid feeding		Paste feeding		Normal feeding	
	HR	*P*	HR	*P*	HR	*P*

Sex	1.044	.950	1.407	.8875	1.306	.6892
*Age*		**.014**	0.978	.7913	0.985	.8415
ASA		.5703		.3855		.6020
ASA II	1.191		1.201		1.104	
ASA III	0.957		1.376		0.854	
Location		.1087		.5515		.2292
Lateral oropharyngeal wall	3.653		3.166		3.187	
Tonsil	4.280		3.224		2.163	
Tongue base	4.120		3.411		2.312	

*T stage*		.2945		**.0417**		**.0108**
T2	0.590		0.68		0.565	
T3	0.69		0.803		0.371	
T4	0.569		0.474		0.309	

*N stage*		**.0187**		.0998		.1647
N1	0.809		0.822		0.812	
N2a	0.41		0.769		0.890	
N2b	0.2986		1.145		1.375	
N2c	0.587		0.493		0.512	
N3	0.537		0.475		0.534	
History of cancer		.690		.4348		.0817

*Induction chemotherapy*	0.451	**.007**	0.547	**.042**	0.544	.057
*Treatment*		**.0003**		.0542		**.0037**
Hyosubglossoepiglottectomy	0.335		1.244		0.290	
Lateral oropharyngectomy	1.209		2.490		1.243	
Exclusive radiotherapy	1.227		2.988		1.017	
Transoral approach	2.44		4.982		1.094	
Mandibular swing	2.943		3.093		1.130	
Reconstructio		.3362		.1374		.0743
Pectoralis major flap	0.882		1.562		2.017	
Sternocleidomastoid flap	3.395		3.896		2.873	
Postoperative complication	1.259	.3623	1.457	.2031	2.028	**.028**
Radiation dose on T	1.012	.7913	1.018	.1417	1.013	.2453
Radiation dose on N	0.986	.119	0.981	**.0444**	0.996	.5902

## Abbreviations

ASA: American Society of Anaesthesiology.
